# Bacterial Profile, Molecular Serotyping, and Key Genetic Determinants for Adhesion, Immune Evasion, and Tissue Spread Among Bulgarian Children with Acute Otitis Media

**DOI:** 10.3390/genes16121512

**Published:** 2025-12-17

**Authors:** Alexandra S. Alexandrova, Vasil S. Boyanov, Raina T. Gergova

**Affiliations:** Department of Medical Microbiology, Medical Faculty, Medical University of Sofia, Zdrave Str. 2, 1431 Sofia, Bulgaria; v.boyanov@medfac.mu-sofia.bg

**Keywords:** genetic determinants for adhesion and tissue spread, molecular serotyping, bacterial spectrum, otitis media

## Abstract

**Background**: Acute otitis media (AOM) is one of the most common pediatric infections. We aimed to investigate the bacterial profile of AOM in children, the serotype distribution, and the main genetic virulence factors involved in adhesion, immune evasion, and tissue spread. **Methods**: In total, 121 AOM cases involving children aged 0 to 14 years were studied. Middle ear fluids (MEF) (n = 42) and nasopharyngeal samples (n = 79) were collected. All strains were identified using routine microbiological tests, conventional PCRs and real-time PCR methods. Molecular serotyping was performed for *S. pneumoniae* and *H. influenzae* isolates. An immunofluorescence serotyping technique was employed for *M. catarrhalis*. Target genetic factors were determined for all involved bacterial agents using singleplex or multiplex PCRs. **Results**: We analyzed 148 nasopharyngeal and MEF. Among 121 AOM cases, a total of 127 bacterial agents were identified, including *S. aureus* (n = 41), *S. pneumoniae* (n = 28), *H. influenzae* (n = 23), *M. catarrhalis* (n = 19), and *S. pyogenes* (n = 16). The leading three serotypes among *S. pneumoniae* were: 19A (18.0%), 6A (14.3%), and 15B (14.3%). 91.3% of *H. influenzae* isolates were non-typeable (lacking a capsule—NTHi). The *M. catarrhalis* isolates were distributed in serotypes A (57.9%), B (26.3%), and C (15.8%). Presence of pili type 1 was detected in 21.4% pneumococci, and the fimbrial gene *hifA* was found in 34.8% of the *H. influenzae* strains. In 73.6% of the *M. catarrhalis* strains, *ompCD* was identified, while 84.2% contained *ompE*. 62.5% of the *S. pyogenes* isolates harbored the *sdc* gene, and 56.2% possessed the *sdaD* gene, predominantly in the MEF isolates. The *cna* adhesin was found in 28.0% of the *S. aureus* strains. **Conclusions**: The monitoring of bacterial pathogens responsible for otitis media, along with their serotype distribution and the prevalence of genetic factors involved in disease pathogenesis, is essential for public health and can help predict disease severity and treatment options.

## 1. Introduction

Acute otitis media (AOM) is among the most prevalent infections in pediatrics, characterized by the presence of fluid inside the middle ear, erythema, and bulging of the tympanic membrane. It is associated with a rapid onset of symptoms such as fever, pain, and irritability. In cases of tympanic membrane perforation, ear discharge may also be noted [1]. In the etiology of AOM, viral and bacterial pathogens are involved, with the most prevalent from the latter category being *Streptococcus pneumoniae*, non-typeable *Haemophilus influenzae*, *Moraxella catarrhalis*, and *Streptococcus pyogenes* (*Streptococcus* group A, GAS) [2,3,4]. The risk of developing AOM is affected by numerous factors. Host factors such as young age, genetic factors, male gender, immunodeficiency, adenoid hypertrophy, and recurrent upper respiratory tract infections are the most common. Environmental factors encompass socio-economic status, attendance at day care facilities such as kindergarten and school, presence of siblings, exposure to tobacco, as well as issues related to overcrowding and access to healthcare [5,6,7]. The factors contributing to the pathogenesis of bacterial OM include Eustachian tube dysfunction and increased bacterial density in the nasopharynx [6].

Many structures of the bacterial cell surface (capsular or slime layer, pili, some outer membrane proteins, and lipopolysaccharides) are included in the mediation of the first step of the infectious process—attachment to mucosal eucaryotic cells—and after that, they promote bacterial colonization and biofilm formation [8,9,10,11]. The subsequent progression of inflammatory response, coupled with the release of inflammatory mediators, determines the clinical symptoms of OM, as well as the onset of complications [12]. Investigating the factors of adhesion is crucial for developing effective prevention and treatment strategies [13]. Furthermore, the expression of these factors and their role in biofilm formation may result in recurrent forms of OM. Their accumulation in combination with invasion factors, produced by more virulent strains, is a cause of the development of complications and severe clinical manifestation of infection [14,15,16]. The pili found in *Streptococcus pneumoniae* (*S. pneumoniae)* are structures that facilitate adhesion, colonization, and biofilm formation. Two distinct types of pili are known, which are encoded by the pathogenic islets PI-1 and PI-2, respectively. The PI-1 genes encode adhesive molecules (*rrgA*, *rrgB*, and *rrgC*), a transcription factor (*rlrA*), and sortase enzymes (*srtB*, *srtC*, and *srtD*), whereas PI-2 consists of genes that encode pilus subunits (*pitA*), a backbone protein (*pitB*), sortase enzymes (*srtG1* and *srtG2*), and a peptidase (*sipA*) [17,18,19]. Previous investigations showed that deleting the *srt* gene from *S. pneumoniae* disturbs the localization of surface proteins and reduces the bacterium’s adherence to human pharyngeal cells in vitro. Experiments conducted using human brain microvascular endothelial cells, brain vascular pericytes, and astrocytes, analyzed through laser scanning confocal microscopy, revealed that the type I/II pili-positive *S. pneumoniae* SP007 strain displayed significantly stronger invasiveness into these cells compared to the pilus-free *S. pneumoniae* ATCC49619 [20,21].

The expression of pili in *Haemophilus influenzae* (*H. influenzae)* is regulated by reversible phase variation, enabling spontaneous shifts between a piliated and a non-piliated state. LKP-type fimbriae are associated with the adhesion of *H. influenzae* to human cells. The *hifA* encodes the major fimbria subunit that mediates binding to a ganglioside receptor [22,23].

The key components of *Moraxella catarrhalis* (*M. catarrhalis)* that promote bacterial colonization and adhesion are the outer membrane proteins CD and E (OMP CD and OMP E) and lipooligosaccharides (LOS). Furthermore, OMP E contributes to the immune response protection, while LOS exhibits endotoxin activity [11,24].

The main factor of virulence in *Streptococcus pyogenes* (GAS) is the M-protein. This surface adhesin is encoded by the *emm* gene, and it is involved in adhesion, immune modulation, and tissue invasion. Additional factors in the pathogenesis of GAS infections include deoxyribonucleases (DNases, encoded by *sdc* and *sdaD*), mainly involved in the degradation of host DNA in neutrophil extracellular traps and the evasion of the immune response [25,26]. The collagen adhesin, synthesized under the control of the *cna* gene, is a member of the *Staphylococcus aureus* (*S. aureus)* microbial surface components recognizing adhesive matrix molecules (MSCRAMM) family. In addition to its primary role in establishing colonization, it also contributes to the inhibition of complement activation, which leads to the evasion of immune responses and promotes the persistence of the infection [27,28].

Molecular serotyping identifies serotypes based on genetic determinants like serotype-specific genes or gene clusters encoding surface antigens. In comparison with conventional, classic serotyping, which relies on agglutination tests using specific antisera and can sometimes display cross-reactivity, molecular serotyping avoids these inaccuracies associated with phenotypic expression [29,30].

**Aim.** This study aims to investigate the bacterial profile of AOM in children, including serotype distribution and genetic factors related to the adherence, immune evasion, and tissue spread of otopathogenic bacteria.

## 2. Materials and Methods

### 2.1. Study Design

We conduct cross-sectional quantitative research on the bacterial spectrum of 121 AOM cases in children, analyzing the distribution of bacterial causative agents, their serotypes, and key genetic determinants involved in the primary steps of AOM pathogenesis.

Inclusion Criteria: The study included patients aged 1 month to 14 years who exhibited typical clinical signs of acute otitis media (AOM) and were examined and diagnosed by a pediatrician or otolaryngologist in a setting between September 2024 and April 2025. During the initial visit for each child, an otoscopist collected either a middle ear fluid (MEF)/otorrhea specimen or a nasopharyngeal specimen, depending on the severity of the case. All MEF and otorrhea specimens that contained bacteria, even in single colonies, were included in the study.

Exclusion Criteria: Children who have initiated antimicrobial therapy and those who have experienced complications in the lower respiratory tract are excluded from this study. Nasopharyngeal specimens with bacterial isolates present at fewer than 10,000 colony-forming units per milliliter (CFU/mL) are also excluded. This is based on the assumption that a lower count of conditionally pathogenic bacteria may indicate they are merely colonizers of the mucous membranes. Once their numbers reach a critical threshold, they may become responsible for causing an infection.

The collected specimens were sent to the microbiological laboratory along with a questionnaire file that included information for the type of specimen, age, sex, history of AOM episodes, attendance at kindergarten/school, number of siblings, presence of allergic rhinitis, and parental cigarette smoking. Written informed consent was obtained from a parent or legal guardian for all children included in the study. Only one episode of AOM was included per patient. Each coinfected bacterium was counted as an independent unit.

### 2.2. Specimen Collection

A total of 148 specimens were collected from children with AOM. The nasopharyngeal samples were collected from children with mild OM cases, without rupture of the tympanic membrane and effusion, but in the presence of symptoms including ear pain, hearing difficulties, fever, irritability, and redness. Middle ear fluids (MEFs) were collected in severe AOM cases during an episode of spontaneous perforation of the tympanic membrane or by tympanocentesis.

The MEF collection was conducted using sterile equipment to prevent contamination of the middle ear with microorganisms. Debris, cerumen, or discharge was removed to allow clear visibility of the tympanic membrane for sampling. The aspiration was performed carefully, avoiding contact with the ear canal in order to minimize the risk of specimen contamination, and allowing for the collection of sufficient fluid for culture and PCR testing.

The specimens were collected using a sterile transport flocked swab (Copan Italia S.p.A., Brescia, Italy) and stored at −4 °C until they could be transported to the microbiological laboratory. The transportation of the specimens was either on the same day of collection or the following day.

### 2.3. Identification of the Bacterial Strains

The specimens were cultivated in Columbia agar with 5% sheep blood, Chocolate agar, McConkey agar, and Sabouraud agar (BD BBL, Becton Dickinson, Franklin Lakes, NJ, USA) at 35 °C, and in the presence of 5–10% CO_2_ for 24–48 h according to the requirements of the suspected isolates.

The presumptive identification was by conventional microbiological tests. The *S. pneumoniae* strains were identified with the optochin test and the bile solubility test. For the *H. influenzae* strains, the oxidase test, Gram stain, satellite growth, requirements on factors X and V, and Remel Rapid NH biochemical identification tests (Thermo Fisher Scientific, Waltham, MA, USA) were performed. The eight biotypes I–VIII of *H. influenzae* were assigned based on the ability of the isolates to produce indole, urease, and ornithine decarboxylase [31]. The *M. catarrhalis* isolates were identified using typical Gram morphology, positive tests for catalase, oxidase, indoxyl acetate esterase reactions, and the hockey puck sign. All *S. pyogenes* isolates were confirmed by colony morphology, beta-hemolysis on blood agar plates, Gram staining, positive pyrrolidonyl-β-naphthylamide (PYR) test, and positive Lancefield group A antigen test (PathoDxtra Strep Grouping Kit, Oxoid™, Thermo Fisher Scientific, Inc., Waltham, MA, USA). *S. aureus* was presumptively identified, showing typical characteristics, including golden yellow color and hemolysis; microscopic morphology of Gram-positive cocci forming grape-like clusters; catalase and coagulase-positive (Rabbit Plasma; Himedia, India) tests. Detailed biochemical identification was made using the Phoenix (BD, BBL, Beckton Dickinson, Franklin Lakes, NJ, USA ).

All strains were identified by both conventional and PCR tests, using specific primers, which allow a reliable diagnosis.

### 2.4. DNA Extraction and PCR Detection

For the extraction of genomic DNA, we used 100 µL of the specimens and the MagCore Genomic DNA Bacterial Kit MagCore^®^ Genomic DNA Bacterial kit (RBC Bioscience Corp., New Taipei City, Taiwan), which allows automated extraction using magnetic-particle technology. The kit is suitable for both Gram-positive and Gram-negative bacteria.

The detection of *H. influenzae, S. pneumoniae,* and *M. catarrhalis*, with the VIASURE real-time PCR kit (Certest Biotec, Zaragoza, Spain) was done following a protocol of initial denaturation at 95 °C for 2 min, and 45 cycles, with denaturation at 95 °C for 10 s, and annealing at 60 °C for 50 s.

Direct PCR-detection of the *S. aureus* isolates was confirmed using primers targeting 23S rRNA genes Sau327 5′-GGACGACATTAGACGAATCA-3′ and Sau1645 5′-CGGGCACCTATTTTCTATCT-3′, resulting in an amplicon size of 1318 bp. DNA was amplified after initial denaturation at 94 °C for 5 min, and 35 cycles of denaturation at 94 °C for 2 min, annealing at 57 °C for 2 min, extension at 72 °C for 1 min, and final elongation at 72 °C for 7 min, as described by Gergova et al. [32].

For the PCR-based detection of *S. pyogenes* (GAS), a multiplex PCR assay was performed using primers for two genes: *speB* (pyrogenic exotoxin B) and *spyCEP* (cell envelope protease). The primer sets used for *speB* were AGACGGAAGAAGCCGTCAGA and TCAAAGCAGGTGCACGAAGC, yielding an amplicon of 952 bp; and GATCCGGCCCATCAAAGCAT and AGCTGCCACTGATGTTGGTG for s*pyCEP*, which produced an amplicon of 786 bp. The PCR amplification was performed with an initial denaturation at 95 °C for 3 min, and 35 cycles of denaturation at 95 °C—35 s; extension at 72 °C—1 min 30 s; annealing at 53 °C for 40 s, and final elongation at 72 °C—7 min, as described by Gergova et al. [33].

### 2.5. Serotyping

Serotyping of *S. pneumoniae* was performed with primer sets according to the CDC protocol https://www.cdc.gov/strep-lab/media/pcr-oligonucleotide-primers.pdf, accessed in 10 June 2025. All serotypes that are co-detected were subjected to ultimate identification in the serogroup. Serotypes 6A and 6C are highly similar in the cps locus. For the specific identification of serotype 6C, we targeted the wciNβ gene, yielding a 359 bp product. The primers used in our study were previously reported by Jin et al. (2009) [34]. The PCR amplification of wciNβ was conducted under the following conditions: 94 °C for 15 s, followed by 35 cycles of 95 °C for 30 s, 60 °C for 60 s, and 72 °C for 60 s, concluding with a final extension at 72 °C for 10 min. For the remaining co-identified serotypes (11A/11D; 15A/15F; 15B/15C; 22F/22A and 24F/24A/24B), serotyping with factor antisera was conducted (Statens Serum Institut, Copenhagen, Denmark). A pure bacterial culture was mixed with the solutions containing latex particles coated with specific antisera. A positive test result was indicated by the agglutination of particles within 10 s, leading to the formation of large visible aggregates.

Serotyping of *H. influenzae*. The PCR-serotyping differentiates the *H. influenzae* strains into encapsulated and non-capsulated isolates (NTHi) based on the presence or absence of the *bex*B gene [35]. The product was processed by an initial denaturation step of 2 min at 95 °C, followed by 30 amplification cycles of 95 °C for 30 s, 54 °C for 30 s, and 72 °C for 45 s. All encapsulated strains were determined into new PCR amplifications with six primer pairs in capsule types “a” to “f”, designed by Falla et al. (1994) [36]. The parameters consisted of 25 cycles of 1 min of denaturation at 94 °C, 1 min of annealing at 60 °C, and 1 min of extension at 72 °C, followed by 10 min at 72 °C.

Serotyping of *M. catarrhalis*. Monoclonal antibodies (MAbs) against the three LOS chemotypes A, B, and C were used for serotyping with an immunofluorescence method. Murine monoclonal antibodies (MAbs), developed in our laboratory using hybridoma technology, were utilized for serotyping [37]. Clones 217H4 and 218A8 are IgG3 isotypes, while clone 219A9 is an IgM isotype. Clone 217H4 reacted exclusively with *M. catarrhalis* serotype A, and clone 218A8 reacted with serotypes A and C. MAb 219A9 recognized a common epitope on the LOS of all three serotypes. The indirect immunofluorescent technique was performed by coating slides with a bacterial suspension, air-drying, and fixing in methanol. Non-diluted MAb supernatants were added and incubated at 37 °C for one hour. Slides were then washed three times with PBS, incubated with FITC-labeled goat anti-mouse antibody for 30 min, and finally examined with a fluorescent microscope (CH30 Olympus).

### 2.6. Detection of Genetic Determinants for Adhesion, Destruction, and Tissue Spread Among Bacterial Isolates from Children with AOM

Detection of type 1 and type 2 pili in *S. pneumoniae*. PCRs were conducted to detect the *rlrA* gene associated with Pilus islet-1 and the *pitB* gene for Pilus islet-2, following the methods described by Aquiar et al. (2008) and Bagnoli et al. (2008) [38,39]. The presence of PI-1 was detected by PCR amplification of an internal fragment of the *rlrA* gene using primers *rlrA*-F (5′-TCTGATAGATGAGACGCTGTTG-3′) and *rlrA*-R (5′-CTCCGCTTCTTTCTACTACAAG-3′) [38]. For the detection of PI-2, primers for PCR amplification of conserved regions within the PI-2 were used as follows: PI-2-F (5′-CGTGGGTATCAGGTGTCCTATG-3′) and PI-2-R (5′-TGCAGTGAATAGCTTTTTAAAGAA-3′). The PCR amplification parameters consist of 35 cycles of denaturation at 94 °C for 15 s, annealing at 60 °C for 15 s, extension at 72 °C for 1 min, and final elongation at 72 °C for 5 min.

Detection of fimbriae in *H. influenzae*. PCR detection was performed to identify the presence of haemagglutinating fimbriae. The target gene was the *hif*A gene encoding the major subunit of haemagglutinating fimbriae in *H. influenzae*. The PCR was carried out by an oligonucleotide primer set: TGCTGTTTATTAAGGCTTTAG and TTGTAGGGTGGGCGTAAGCC, described previously by Geluk et al. (1998) [22] in a program of 30 cycles of 95 °C for 1 min, 55 °C for 1 min, 72 °C for 2 min, and final elongation at 72 °C for 8 min. Detection of outer membrane proteins in *M. catarrhalis*. In *M. catarrhalis* strains, we examined the outer membrane proteins OMP CD (5′-GTGTGACAGTCAGCCCACTA-3′ and 5′-TTGCTACCAGTGATTACTGA-3′) and OMP E (5′-TTCAACCCTAACCGCAAC-3′ and 5′-TTTGGCGTGATAAGCAAG-3′) with the conditions described previously by Mitov et al. (2010) [14]. The pairs of primers for OMP were amplified in 25 cycles under the following conditions: 30 s at 95 °C, 1 min at 58 °C, and 2 min 30 s at 72 °C, yielding PCR products of 1200 bp for *ompCD* and 1300 bp for *ompE*.

Detection of collagen-binding protein in *S. aureus*. The collagen-binding protein in *S. aureus* is encoded by *the cna* gene, and it was detected by PCR amplification with primer set AAAGCGTTGCCTAGTGGAGA and AGTGCCTTCCCAAACCTTTT, and parameters as follows: 30 s at 95 °C, 1 min at 66 °C, and 2 min at 72 °C, resulting in a 192 bp amplicon [40]. Detection of deoxyribonucleases in *S. pyogenes*. The gene *sdc* (streptodornase C or deoxyribonuclease C) was amplified with primer set AAGCTTAGAAACTCTCTCGCCA and AGTTCCAGTAATAGCGTTTTTCCGT, resulting in an amplicon size of 600 bp. The primer set used for the amplification of gene *sdaD* encoding the extracellular deoxyribonuclease (DNase) was TTTACGCTGAATCGGGCACT and GGCTCTGGTTTGCTTTCCCA, yielding an amplicon of 295 bp. The cycling conditions included initial denaturation at 94 °C for 5 min, and 30 cycles: at 94 °C for 30 s, 60 °C for 30 s, 72 °C for 30 s, and 72 °C for 5 min, described by Gergova et al. (2019) [15].

### 2.7. Statistical Analysis

Statistical analyses were conducted using IBM SPSS Statistics for Windows v19.0 (IBM Corp., Armonk, NY, USA).

## 3. Results

### 3.1. Studied Population

Our study involved 148 cases, of which 121 cases (81.7%) were positive for pathogenic bacteria using direct PCR methods, and 119 cases (80.4%) were culture-positive. The results from the PCR identification methods and the conventional microbiological identification tests showed very high similarity (98.3%). Only two nasopharyngeal specimens were culture-negative, but the PCR results revealed the presence of bacterial DNA. The remaining 27 cases (18.3%) showed no evidence of bacterial or fungal growth. The demographic data of the 121 children with otitis media are presented in Table 1. The study was conducted on 65.3% nasopharyngeal specimens (n = 79) and 34.7% middle ear fluids/otorrhea specimens (n = 42). Demographic data is presented in Table 1. Clinical and environmental factors associated with the studied AOM cases are summarized in Table 2.

Statistical significance was observed with a *p*-value of less than 0.05 for the following factors: having siblings, exposure to parental smoking, and the presence of allergic rhinitis in patients.

The majority of patients experienced a first episode of AOM, accounting for 92.5%. Approximately 90.0% of the children attended kindergarten or school. Less than half of the patients (36.3%) reported having siblings. For the small number of patients, the parents declared that they suffer from allergic rhinitis (20.6%). A significant number of the patients were exposed to passive smoking. We measured the interactions between pairs of environmental factors in relation to disease development using the Case-only odds ratio (CO-OR). For all interactions analyzed, we found a positive interaction indicating a synergistic effect, with CO-OR values greater than 1.

CO-OR kindergarten/brothers–sisters =1.91
CO-OR kindergarten/smoking = 4.98
CO-OR brothers–sisters/smoking = 1.06

### 3.2. Bacterial Investigation

**Figure 1:**
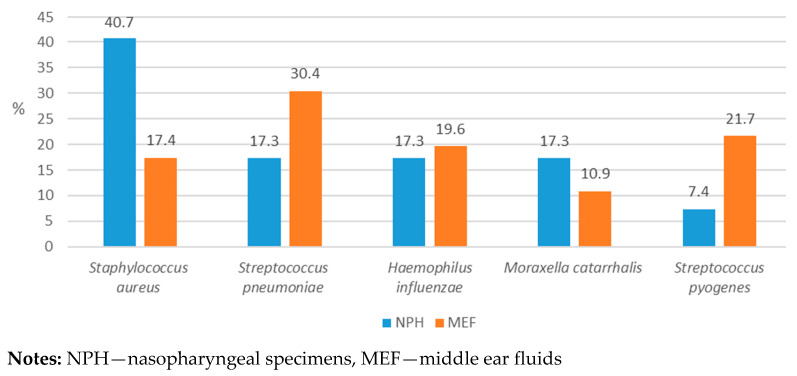
Distribution of bacterial isolates among nasopharyngeal and middle ear fluids collected from patients with OM.

Among all 121 AOM cases, a total of 127 bacterial pathogens were identified, including *S. aureus* (32.3%, n = 41), *S. pneumoniae* (22.0%, n = 28), *H. influenzae* (18.1%, n = 23), *M. catarrhalis* (15.0%, n = 19), and *S. pyogenes* (12.6%, n = 16) (Figure 1).

**Figure 2:**
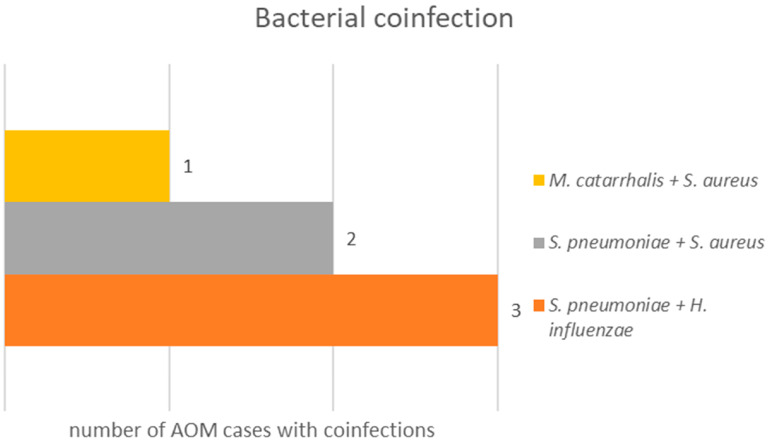
Bacterial co-infections among 121 acute otitis media (AOM) cases in children.

In six children, more than one bacterial representative was found, illustrated in Figure 2.

Among the six identified co-infections, five occurred in children aged between 2 and 6 years, and one case was in a 9-year-old child. In terms of sex distribution, four of the affected children were males, and two were females. All children had siblings, were immunized, and were exposed to parental smoking. One child had a history of allergic rhinitis.

### 3.3. Molecular Serotyping

#### 3.3.1. Serotyping of *S. pneumoniae*

**Figure 3:**
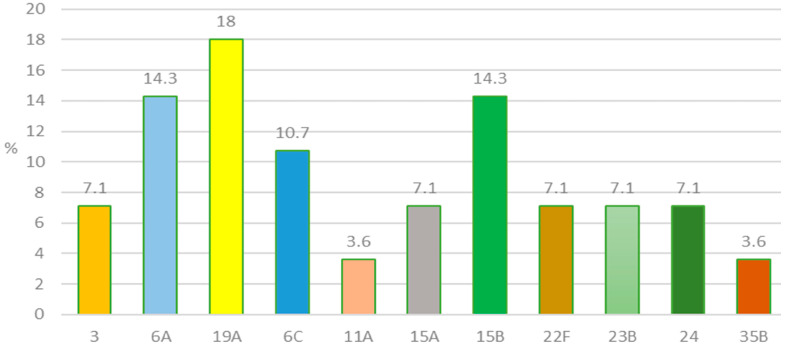
Serotypes among *S. pneumoniae* isolates recovered from children with acute otitis media (2024–2025).

**Figure 4:**
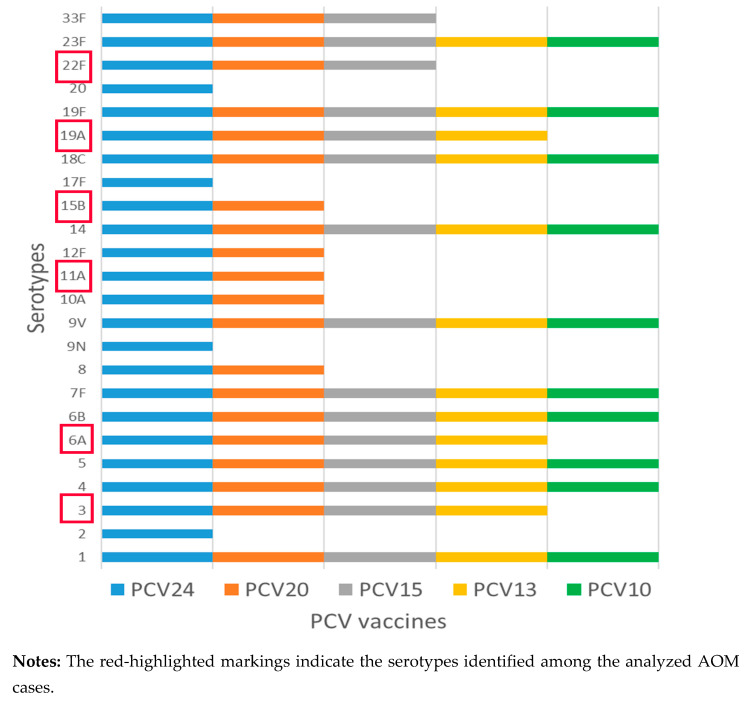
Serotypes in available pneumococcal conjugate vaccines (PCVs)—formulations.

The serotyping of *S. pneumoniae* disclosed eleven distinct serotypes, illustrated in Figure 3. The predominant serotypes were 19A (18.0%), 6A (14.3%), 15B (14.3%), and 6C (10.7%) (Figure 3). All recognized serotypes are non-vaccinal for our country. PCV10 vaccine is included in the Bulgarian mandatory immunization program, and it does not cover these serotypes. Figure 4 lists the serotypes included in the available pneumococcal conjugate vaccines worldwide.

The predominant serotypes 19A, 6A, 15B and 6C accounted for 57.1% of all recognized serotypes among the 28 *S. pneumoniae* AOM isolates. They were distributed in the examined age groups as follows: 0–2 years of age (18.8%), 3–6 years of age (62.5%), 7–14 years of age (18.8%).

Both the PCV13 and PCV15 vaccines covered 39.2% of these serotypes. The PCV20 and PCV24 vaccines showed coverage for 57.1% of the identified serotypes. The serotypes that do not fall under any of the available pneumococcal conjugate vaccines were 35.7%.

#### 3.3.2. Serotyping and Biotyping of *H. influenzae*

**Figure 5:**
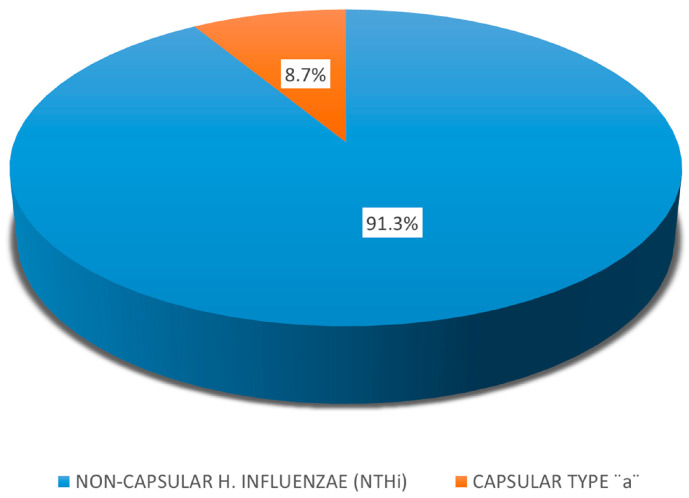
Distribution of capsular and non-capsular types of 23 *H. influenzae* isolates among children with AOM (2024–2025).

Capsular typing of *H. influenzae*. The typing of *H. influenzae* disclosed that 91.3% of the strains were non-capsular (NTHi) based on the absence of the *bexb* gene, which is the major gene for the export and expression of its capsule. Only two strains are affiliated with serotype “a”(Figure 5).

**Figure 6:**
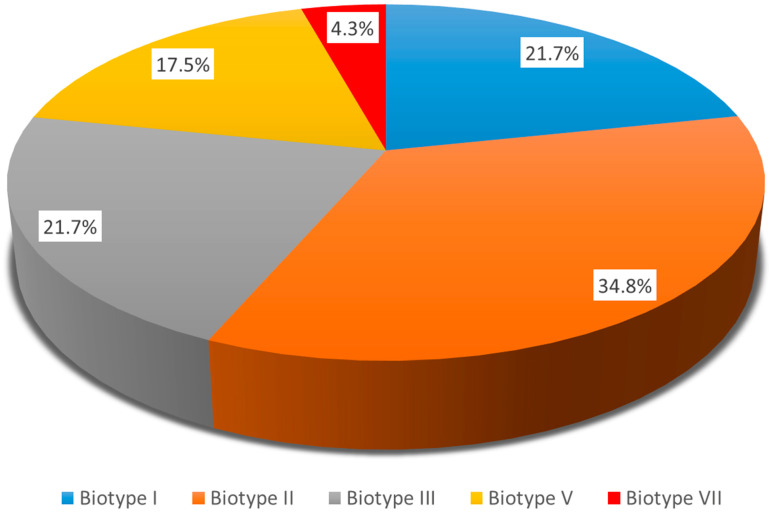
Biotypes of *H. influenzae* strains isolated from children with acute otitis media.

Biotyping of *H. influenzae*. The biotyping distributed the *H. influenzae* strains into five biotypes. The most predominant were Biotype II, followed by Biotype I, Biotype III, Biotype V, and Biotype VII, illustrated in Figure 6.

#### 3.3.3. Serotyping of *M. catarrhalis*

**Figure 7:**
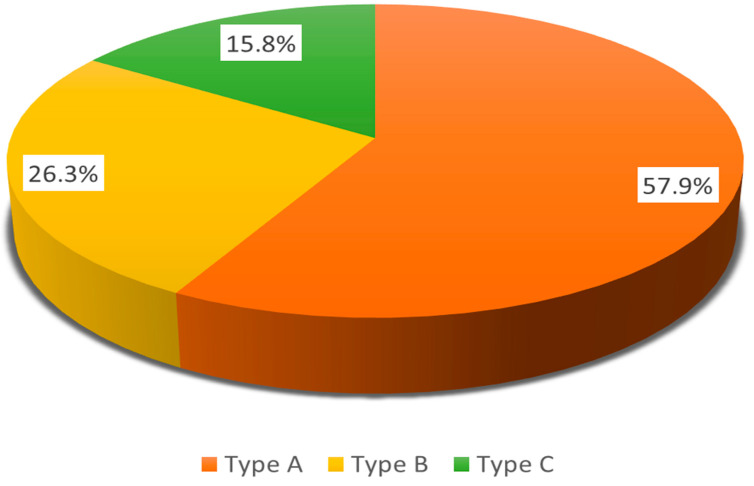
Distribution of serotypes among 19 *M. catarrhalis* isolates from children with acute otitis media.

The serotyping of *M. catarrhalis* strains was performed using immunofluorescence with MAbs and revealed that serotype A is the most common among the tested strains (57.9%, n = 11), followed by serotype B (26.3%, n = 5) and serotype C (15.8%, n = 3) (Figure 7).

The predominant serotype A was primarily found in the age group of 3 to 6 years, accounting for 63.6% of cases, followed by 27.3% in the 7 to 14 years age group, and one isolate identified in a 1-year-old child. Serotypes B and C were detected in single isolates from various age groups. We did not observe an association between the distribution of serotypes and the age of the patients.

### 3.4. Detection of Virulence Factors Associated with Adhesion, Immune Evasion, and Tissue Spread Among the Studied Bacterial Population from Children with OM

A total of 69.6% of the bacterial agents recovered from MEF isolates carried genes for adhesion, biofilm formation, and/or invasion. Among the nasopharyngeal isolates, 53.0% of the otopathogens showed the presence of the examined virulence factors.

#### 3.4.1. Presence of Pili in *S. pneumoniae*

**Figure 8:**
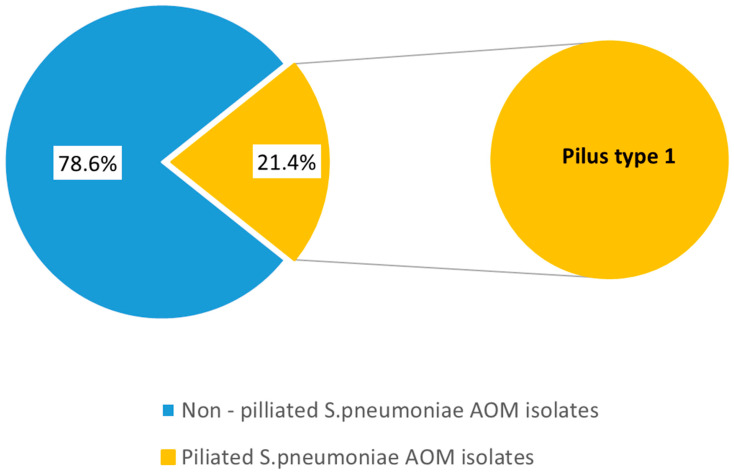
Distribution of pili among 28 *S. pneumoniae* isolates recovered from children with AOM.

Pili were detected in 21.4% (n = 6) of the examined pneumococcal strains. PCR analysis revealed the presence of the *rlrA* gene, which is associated with Pilus islet-1 (Figure 8). Pilus islet-2 was not found in the studied population. The distribution of pilliated strains among serotypes was as follows: 19A (n = 2), 6A (n = 2), 6C (n = 1), and 23B (n = 1). Among the nasopharyngeal samples, two pilliated pneumococcal strains were identified, with the majority found in MEF samples (n = 4). All pilliated strains were isolated from children under the age of 7 (n = 6).

#### 3.4.2. PCR Detection of Fimbriated *H. influenzae* Isolates

The fimbrial typing was carried out for all encapsulated and non-capsulated variants. Haemagglutinating fimbriae were detected in eight isolates (34.8%), recovered from five MEF isolates and three nasopharyngeal specimens. Fimbrial gene *hif*A was detected only in NTHi strains. Of the fimbriated isolates, six belonged to biotype II, while two were from biotype I.

#### 3.4.3. Detection of Streptodornases C and D, Promoting Invasion, Among *S. pyogenes* Strains

The *sdc* gene was found in 62.5% of the *S. pyogenes* isolates. The majority of these strains (80.0%) were isolated from MEF samples. The *sdaD* gene was detected in 56.2% of the isolates, and 77.7% were isolated again from MEF samples; the rest were found among nasopharyngeal strains.

#### 3.4.4. Detection of OMP CD and OMP E Surface-Exposed Outer Membrane Adhesins Among *M. catarrhalis*

The PCR results revealed the presence of *ompE* and *ompCD* genes in 84.2% and 73.6% of the *M. catarrhalis* strains distributed only in nasopharyngeal samples from all three serotypes A, B, and C.

#### 3.4.5. Detection of the *cna* Gene in *S. aureus*

The collagen adhesin protein, encoded by the *cna* gene, was detected in 29.2% of the isolates, distributed among eight strains isolated from MEF samples and four recovered from nasopharyngeal samples.

The identified genetic determinants among the studied strains are illustrated in Table 3.

The statistical comparison of the distribution of the studied genetic determinants among the bacterial agents and the comparison between the types of the collected specimens disclosed statistical significance at *p*-value < 0.05 for the DNAases in *S. pyogenes*, and non-significant results for the presence of pili in *S. pneumoniae* and *H. influenzae*. Nevertheless, the percentage distribution among all tested isolates of *S. pneumoniae*, *H. influenzae*, and *S. pyogenes* indicated a higher frequency of the genetic determinants in the MEF samples. Overall, the statistical comparison of the gene frequencies in the MEF isolates (76.2%) versus nasopharyngeal isolates (54.4%) revealed significant results at *p* = 0.030 for the distribution of the studied genetic determinants among the MEF isolates.

The statistical analysis with confidence intervals (CIs) revealed non-significant results, as the null hypothesis is not rejected, for the distribution of pili among the nasopharyngeal and MEF specimens recovered from pneumococci. The result for fimbriae in *H. influenzae* was not statistically significant as well, as the 95% CI included the null value.

The CI suggests a clinically meaningful effect for the distribution of DNases in *S. pyogenes*. It is significantly greater for MEF isolates compared to their distribution in nasopharyngeal samples. Regarding the *cna* gene identified in nasopharyngeal and MEF *S. aureus* isolates, the result is not statistically significant.

## 4. Discussion

In our study, we identified the bacterial profile of acute otitis media (AOM) in children up to 14 years old. Most of the affected children were between 3 and 6 years of age, with both females and males represented. AOM is particularly prevalent in young children due to their anatomical and immune system factors. It is the most common complication of rhinopharyngitis and adenoiditis in children under the age of five, largely because of dysfunction in the Eustachian tube. The causes of AOM include both viruses and bacteria. Viral infections of the upper respiratory tract often occur prior to bacterial AOM, creating an environment that facilitates bacterial overgrowth [1,4,6]. In both nasopharyngeal and middle ear fluids, we found the most prevalent bacteria, cited in different investigations worldwide [3,41]. According to our data, we may summarize that the prevalence of *S. pneumoniae*, *S. pyogenes*, and *H. influenzae* isolates is associated mostly with AOM in children with tympanic membrane perforation and spontaneous otorrhea, where the most common identified bacteria from mild AOM cases were *S. aureus*, *S. pneumoniae*, *H. influenzae*, and *M. catarrhalis*.

Most of the severe AOM cases occurred in the same age group (3–6 years of age), predominantly among males. Regarding the associated etiological and risk factors for otitis media, attending kindergarten and exposure to passive smoking are thought to be the most important risk factors for OM, followed by having siblings. All these factors are linked to the formation of collective immunity in early childhood and a potentially weakened immune system [7].

We used molecular serotyping for the capsular antigens of *S. pneumoniae* and *H. influenzae* strains. The capsule is the primary virulence factor in both pneumococci and *H. influenzae*. It enables the bacterium to evade host immune responses, particularly phagocytosis and complement-mediated killing, and plays a role in adhering to epithelial surfaces. More than 100 *S. pneumoniae* capsular serotypes have been identified, but a limited number of them are responsible for invasive diseases and are covered by the available PCVs [42,43].

Almost all children in our study are immunized according to the mandatory immunization schedule in our country, including PCV10 for *S. pneumoniae* and Hib in the combined vaccine against diphtheria, tetanus, pertussis, polio, and hepatitis B. Fifteen years after the introduction of PCV10 in our country, we observe a dramatic reduction in the incidence of invasive pneumococcal diseases [44]. However, *S. pneumoniae* remains a leading pathogen in OM cases, specifically with non-vaccinal serotypes. All recognized serotypes in our study were non-vaccinal for our country and predominantly found among children under six years of age. The predominance of serotypes 19A, 6A, and 6C has been discussed in numerous investigations as leading serotypes in different groups of patients with both invasive and non-invasive diseases [45,46,47]. Most of the 19A isolates were reported to be multidrug-resistant clones, clustered in widely spreading clones, like CC320, which is predominant in our country [44]. Multidrug-resistant pathogens can form biofilms in ear tissue, reducing antibiotic effectiveness and presenting significant treatment challenges [48,49]. Serogroup 6 has changed due to capsular switch processes. Serotype 6B was the dominant serotype before the introduction of PCV10 in our country, but now serotypes 6A and 6C prevail. Serotype 6C is also distributed globally, described in many studies from various geographic areas, and it is not included in the formulations of the available pneumococcal vaccines. An emerging serotype in our study was 15B, which ranks as the second most prevalent serotype, and is included in the formulations of PCV20 and PCV24 [50]. We did not find any associations between specific serotypes and disease severity. All serotypes were present in both nasopharyngeal and MEF samples, collected from cases of mild and severe AOM, respectively.

The non-vaccine serotypes of *S. pneumoniae* can have significant clinical and public health implications. The incidence of these non-vaccine serotypes has increased due to serotype replacement, leading to their successful spread across various geographic areas. The non-vaccine serotypes can vary in terms of virulence, clinical severity, and patterns of resistance [51]. They may influence the distribution of serotypes among nasopharyngeal carriage, as well as non-invasive and invasive disease. The reduced impact of the vaccines may affect herd immunity, antimicrobial resistance, and disease prevention efforts [44,50,51]. The monitoring of serotype-specific trends in antimicrobial resistance and virulence is crucial for public health surveillance. Newly emerging serotypes may lead to an increase in disease cases and could require new strategies for empirical therapy.”

The capsular serotypes of *H. influenzae* vary from “a” to “f”. The non-typeable *H. influenzae* strains lack a capsule and are common colonizers of the upper respiratory tract and major causes of otitis media and sinusitis [52,53]. In the post-Hib vaccine era, infections with other capsulated types, including *H. influenzae* types “a” (Hia) and “f” (Hif), are emerging. Infections due to non-type “b” and NTHi have become relatively more common [52,54,55]. Almost all identified *H. influenzae* strains in our study were non-capsular. NTHi appeared as a leading AOM pathogen, found both in mild and severe AOM cases. The non-typeable *H. influenzae* strains can attach to various host proteins, both by adhering to the surface of epithelial cells and by capturing serum factors. These binding interactions assist NTHi to develop a stronger adhesive presence on host cells to mediate colonization or to provide defensive mechanisms like an evasion of the host immune response or biofilm formation. The biotyping reveals an association with particular clinical diagnoses. The biotypes, predominantly associated with OM among children in our study, were Biotype II, followed by Biotype I and Biotype III.

The development of respiratory tract infection, and particularly AOM, is preceded by nasopharyngeal colonization. The transmission, colonization, and invasion of the pathogens depend on their ability to avoid the host’s inflammatory and immune responses [51,56]. In our research, we examined important virulence factors associated with the adhesion, destruction, immune evasion, and tissue spreading as critical steps of otitis media pathogenesis. The pilus plays a significant role in tissue tropism, biofilm formation, modulation of innate immune responses, and overall contribution to virulence. The role of the pilus is crucial for adhesion to the extracellular matrix proteins in the host, evading mucosal clearance, which is extensively discussed in various studies [57,58,59]. The pneumococcal pili are highly immunogenic structures under the selective pressure of the host’s immune responses. Among the pneumococcal strains, we identified only the pili of PI-1 type. PI-2 adhesins were not detected. Studies demonstrated that Pilus type-2 varies from 0% to 21% in invasive diseases, otitis media, and carriers [19,57]. The PI-1 adhesin is not uniformly present among the different pneumococcal serotypes. Serotypes 19A and 6C were found in the studied pilliated strains. The presence of Pili type I was not strictly associated with mild or severe AOM cases.

Fimbriae were detected in less than half of the studied *H. influenzae* strains, mostly from nasopharyngeal samples, collected from children with mild AOM cases. There are reports that non-fimbriated strains also showed an ability to attach to mucosal cells. The fimbriae exhibit functions similar to *S. pneumoniae* pili and are related to the onset and persistence of infections [23,60].

*M. catarrhalis* strains were mostly represented by serotype A, which was predominantly found in cases of mild otitis media. Compared with the results of an earlier study of Bulgarian *M. catarrhalis* isolates from patients with various respiratory tract infections and different ages, serotype A is still the leading serotype in our country [61].

Among the *M. catarrhalis* strains, we found that a significant number of them exhibited the presence of *ompCD* and *ompE* genes, recovered from patients with OM without effusion. OMP CD, as a major virulence-associated surface protein, was very common among the otopathogenic isolates. The increased virulence of certain *M. catarrhalis* strains is mainly due to the expression of the *ompE* and *ompCD* genes and the presence of intact LOS. These factors play a crucial role in the initial attachment to human mucosal epithelial cells and survival in biological fluids. The *ompE* and *ompCD* genes encode porins that function as adhesion molecules, mediate nutrient transport, fatty acids accumulation, and a complement-resistance of *M. catarrhalis*, and are essential to serum resistance [14,62,63,64,65]. The presence of OMP CD significantly increases the ability of *M. catarrhalis* to bind to respiratory epithelial cells, especially to human nasopharyngeal, middle-ear mucins, and human lung. OMP CD contributes to the bacterium’s resistance to adverse conditions, especially serum complement, and is highly associated with respiratory tract infections. This antigen is a potential vaccine candidate. Antibodies to the potential vaccine antigen, OMP CD, inhibit binding to mucin. *M. catarrhalis* surface molecules are critical for complement resistance, but they are also targeted by antibodies that subsequently trigger complement activation. Studies demonstrated that strains lacking OMP E appeared to be more sensitive to serum-mediated killing [11,63,66,67,68].

*S. pyogenes* (GAS) components, including the hyaluronic acid capsule, fimbriated structures, M proteins, and the fibronectin-binding adhesin, contribute to adhesion and colonization of the pathogen in the nasopharynx region, including tonsil lymphoid tissue [13,25,26]. Once colonized within the infected place, GAS disseminates inside the host by surviving and multiplying. GAS survives by different mechanisms, including hiding within the epithelial cell lines, inhibiting phagocytosis, and degrading the cells by DNases that destroy the DNA backbone of neutrophil extracellular traps, allowing GAS to avoid neutrophil killing. GAS-infected cells trigger a strong inflammatory response, thereby inducing a cytokine storm [69,70].

The *sdc* gene was found in more than half of the *GAS* isolates. Most of the strains that carried *sdc* were associated with the severe cases of OM, accompanied by perforation of the tympanic membrane. There are other reports that highlighted the important biological role of the DNases in facilitating the bacterial spread in necrotic sites and their association with invasive infections [25,71,72]. SdaD possesses a similar biological role, allowing the bacteria to escape innate immune defenses, evade neutrophils, and play a supporting role in systemic dissemination [72,73]. More than half of the studied strains possessed DNase D and were found predominantly in patients with effusion.

Thacharodi et al. (2024) reported that GAS accounts for 14% of hospitalized heavy cases of OM, while it is believed to cause only 2–3% of cases in other children with non-severe OM [70]. Some GAS infections cause serious cases of neutropenia, after degradation of leucocytes by DNases, which results in poor patient prognosis. Further, the administration of a neutrophil-depleting antibody has been studied to change GAS infection from a non-invasive to an invasive form in mouse models [70]. One-third of the Bulgarian otopathogenic *S. aureus* isolates carried the *cna* gene, predominantly in cases of severe AOM. It not only aids colonization but also inhibits complement activation, allowing the infection to evade immune responses and persist longer.

Research by Madani et al. (2017) provides insight into the structural and conformational dynamics of CNA and its interactions with collagen [27]. The findings indicate that the linker region of CNA and specific residues within it are essential for the formation of the CNA-collagen complex. CNA and similar adhesins may preferentially bind to sites where collagen fibers have been cleaved, such as in wounded, damaged, or inflamed tissues, or in areas where collagen is less mature [27].

The limitations of the study include a relatively small number of middle ear fluids analyzed, which was determined by the severity of the cases and the study period being limited to one winter-spring season. The study period is limited (September 2024–April 2025), which reduces the epidemiological strength of the study. Despite the small sample size, our research provides a clear overview of the primary bacterial agents responsible for acute otitis media, their serotype distribution, and the presence of key genetic determinants that encode various virulence factors.

## 5. Conclusions

The prevalent risk factors observed in our study for the development of otitis media are preschool age, attendance at kindergarten, and exposure to passive smoking. The molecular serotyping revealed that the studied pediatric population consisted mostly of non-vaccinal pneumococci, NTHi, and serotype “A” *M. catarrhalis* otopathogenic bacteria. Around 70% of the middle ear fluid isolates carried genes for adhesion, biofilm formation, and/or invasion, which significantly enhance their pathogenicity and capacity to cause severe diseases. The monitoring of bacterial pathogens responsible for otitis media, along with their serotype distribution and the prevalence of genetic factors involved in disease pathogenesis, is essential for public health and can help predict disease severity and treatment options.

## Figures and Tables

**Table 1:** Demographic data of 121 pediatric cases of AOM * between September 2024 and April 2025.

Sex (n, %)	Age (n)	Sample Collection (n)
**Male (n = 73, 60.3%)**	0–2 (n = 7) 3–6 (n = 48) 7–14 (n = 18)	**Nasopharyngeal sample (n = 48)**
		**Middle ear fluid (n = 25):** Tympanocentesis (6); Spontaneous perforation (19)
**Female (n = 48, 39.6%)**	0–2 (n = 2) 3–6 (n = 34) 7–14 (n = 12)	**Nasopharyngeal sample (n = 31)**
		**Middle ear fluid (n = 17):** Tympanocentesis (5); Spontaneous perforation (10)
**Total (100%)**	**n = 121**	**Nasopharyngeal samples = 79; Middle ear fluids= 42**

**Notes**: * AOM—acute otitis media.

**Table 2:** Clinical and environmental factors associated with 121 *AOM cases among children.

Factors Associated with AOM *	Age of the Patient			Total n (%)	*p*-Value ** 0–6 y/7–14 y
	0–2 Years n (%)	3–6 Years n (%)	7–14 Years n (%)		
**First episode of AOM**	9 (100)	76 (92.6)	27 (90.0)	112 (92.5)	0.688
**Attendance in kindergarten/school**	0 (0)	78 (95.1)	30 (100)	108 (89.2)	-
**Brothers/sisters**	4 (44.4)	21 (25.6)	19 (63.3)	44 (36.3)	0.0008
**Allergic rhinitis**	0 (0%)	12 (14.6)	13 (43.3)	25 (20.6)	0.001
**Parental cigarette smoking**	5 (55.5)	46 (56.1)	24 (80.0)	76 (61.9)	0.029
**Total n of AOM cases**	9	82	30	121 (100%)	

Notes: * AOM—acute otitis media, n—number. ** a *p*-value < 0.05 is considered statistically significant.

**Table 3:** Distribution of main genetic determinants for adhesion, immune evasion, and tissue spread among bacterial agents associated with AOM in children.

Bacterial Agent	Gene	Product	Function	Total n (%) of Genes	Distribution Among Specimens		*p*-Value ^3^ and 95% CI ^4^ (NPH/MEF)
					NPH ^1^	MEF ^2^	
***S. pneumoniae*** **n = 28**	*rlrA*	Pili Type I	Adhesion and colonization	6 (21.4)	2 (7.1)	4 (14.3)	0.392 [−0.862, 0.342]
***H. influenzae*** **n = 23**	*hifA*	Fimbriae	Adhesion and colonization	8 (34.8)	3 (13.0)	5 (21.8)	0.275 [−0.697, 0.177]
***S. pyogenes*** **n = 16**	*sdc*	DNase C	Destruction, immune evasion, tissue spread	10 (62.5)	2 (12.5)	8 (50.0)	0.015 [−0.467, −0.053]
	*sdaD*	DNase D	Destruction, immune evasion, tissue spread	9 (56.2)	2 (12.5)	7 (43.7)	0.033 [−0.499, −0.021]
***S. aureus*** **n = 41**	*cna*	Collagen adhesin protein	adherence, immune evasion	12(29.2)	4 (9.7)	8 (19.5)	0.109 [−0.578, 0.058]
***M. catarrhalis*** **n = 19**	*ompCD*	Outer membrane protein CD	Porin activity, immune evasion, adhesion	14 (73.6)	14 (100)	0 (0)	-
	*ompE*	Outer membrane protein E	surface-exposed adhesin, immune evasion	16 (84.2)	16 (100)	0 (0)	-

**Legend**: ^1^ NPH—nasopharyngeal specimen. ^2^ MEF—middle ear fluid. ^3^ a *p*-value < 0.05 is considered statistically significant. ^4^ CI—confidence interval.

## Data Availability

All datasets generated or analyzed during the study are included in the manuscript.

## References

[1] Dermawan A., Ropii B., Lasminingrum L., Hasansulama W., Setiabudiawan B. (2025). Determinants of acute otitis media in children: A case-control study in West Java, Indonesia. Medicina.

[2] Sillanpää S., Oikarinen S., Sipilä M., Kramna L., Rautiainen M., Huhtala H., Aittoniemi J., Laranne J., Hyöty H., Cinek O. (2016). *Moraxella catarrhalis* might be more common than expected in acute otitis media in young finnish children. J. Clin. Microbiol..

[3] Imöhl M., Perniciaro S., Busse A., van der Linden M. (2021). Bacterial spectrum of spontaneously ruptured otitis media in a 7-Year, longitudinal, multicenter, epidemiological cross-sectional study in Germany. Front. Med..

[4] Gattinara C.G., Bergamini M., Simeone G., Reggiani L., Doria M., Ghiglioni D.G., Terminiello A., Cosentino F., Cursi L., Donà D. (2025). Antibiotic treatment of acute and recurrent otitis media in children: An Italian intersociety Consensus. Ital. J. Pediatr..

[5] Kong K., Coates H.L. (2009). Natural history, definitions, risk factors and burden of otitis media. Med. J. Aust..

[6] Schilder A.G., Chonmaitree T., Cripps A.W., Rosenfeld R.M., Casselbrant M.L., Haggard M.P., Venekamp R.P. (2016). Otitis media. Nat. Rev. Dis. Primers.

[7] Folino F., Bosi P., Torretta S., Gaffuri M., Marchisio P. (2022). Recurrent acute otitis media environmental risk factors: A literature review from the microbiota point of view. Appl. Microbiol..

[8] Kirkeby S., Friis M., Mikkelsen H.B., Cayé-Thomasen P. (2011). Bacterial adherence in otitis media: Determination of N-acetylgalactosamine (GalNAc) residues in the submucosal glands and surface epithelium of the normal and diseased Eustachian tube. Microb. Pathog..

[9] van den Broek M.F.L., De Boeck I., Kiekens F., Boudewyns A., Vanderveken O.M., Lebeer S. (2019). Translating recent microbiome insights in otitis media into probiotic strategies. Clin. Microbiol. Rev..

[10] Massa H.M., Spann K.M., Cripps A.W. (2021). Innate immunity in the middle ear mucosa. Front. Cell. Infect. Microbiol..

[11] Zahid A., Wilson J.C., Grice I.D., Peak I.R. (2024). Otitis media: Recent advances in otitis media vaccine development and model systems. Front. Microbiol..

[12] Juhn S.K., Jung M.K., Hoffman M.D., Drew B.R., Preciado D.A., Sausen N.J., Jung T.T., Kim B.H., Park S.Y., Lin J. (2008). The role of inflammatory mediators in the pathogenesis of otitis media and sequelae. Clin. Exp. Otorhinolaryngol..

[13] Muhtarova A.A., Boyanov V.S., Alexandrova A.S., Gergova R.T. (2025). Molecular characterization of *Streptococcus pyogenes* isolates recovered from hospitalized patients during the years 2023–2024. Microorganisms.

[14] Mitov I.G., Gergova R.T., Ouzounova-Raykova V.V. (2010). Distribution of genes encoding virulence factors *ompB2*, *ompCD*, *ompE*, β-lactamase and serotype in pathogenic and colonizing strains of *Moraxella catarrhalis*. Arch. Med. Res..

[15] Gergova R., Muhtarova A., Mitov I., Setchanova L., Mihova K., Kaneva R., Markovska R. (2019). Relation between *emm* types and virulence gene profiles among Bulgarian *Streptococcus pyogenes* clinical isolates. Infect. Dis..

[16] Khan R.M.A., Anwar S., Pirzada Z.A. (2020). *Streptococcus pyogenes* strains associated with invasive and non-invasive infections present possible links with *emm* types and superantigens. Iran J. Basic. Med. Sci..

[17] Ness S., Hilleringmann M. (2021). *Streptococcus pneumoniae* type 1 pilus—A multifunctional tool for optimized host interaction. Front. Microbiol..

[18] Alexandrova A.S., Pencheva D.R., Setchanova L.P., Gergova R.T. (2022). Association of pili with widespread multidrug-resistant genetic lineages of non-invasive pediatric *Streptococcus pneumoniae* isolates. Acta Microbiol. Immunol. Hung..

[19] Miao C., Cui Y., Yan Z., Jiang Y. (2023). Pilus of *Streptococcus pneumoniae*: Structure, function and vaccine potential. Front. Cell. Infect. Microbiol..

[20] Paterson G.K., Mitchell T.J. (2006). The role of *Streptococcus pneumoniae* sortase A in colonisation and pathogenesis. Microbes Infect..

[21] Chen S.-N., Wang Y.-J., Li M.-J., Xing Y.-Q., Guo M.-Y., Yan J., Sun A.-H. (2025). Type I and II pili mediate *Streptococcus pneumoniae* invasion into human blood-brain barrier-derived cells through extracellular matrix protein receptors and different endocytosis pathways. Int. Immunopharmacol..

[22] Geluk F., Eijk P.P., van Ham S.M., Jansen H.M., van Alphen L. (1998). The fimbria gene cluster of nonencapsulated *Haemophilus influenzae*. Infect. Immun..

[23] Häuser S., Wegele C., Stump-Guthier C., Borkowski J., Weiss C., Rohde M., Ishikawa H., Schroten H., Schwerk C., Adam R. (2018). Capsule and fimbriae modulate the invasion of *Haemophilus influenzae* in a human blood-cerebrospinal fluid barrier model. Int. J. Med. Microbiol..

[24] Anderson T., Jiang H., Cheallaigh A.N., Bengtsson D., Oscarson S., Cairns C., St Michael F., Cox A., Kuttel M.M. (2024). Formation and immunological evaluation of *Moraxella catarrhalis* glycoconjugates based on synthetic oligosaccharides. Carbohydr. Polym..

[25] Chen M., Wang W., Tu L., Zheng Y., Pan H., Wang G., Chen Y., Zhang X., Zhu L., Chen J. (2017). An *emm*5 group A streptococcal outbreak among workers in a factory manufacturing telephone accessories. Front. Microbiol..

[26] Gergova R., Boyanov V., Muhtarova A., Alexandrova A. (2024). A Review of the impact of streptococcal infections and antimicrobial resistance on human health. Antibiotics.

[27] Madani A., Garakani K., Mofrad M.R.K. (2017). Molecular mechanics of *Staphylococcus aureus* adhesin, CNA, and the inhibition of bacterial adhesion by stretching collagen. PLoS ONE.

[28] Foster T.J. (2019). The MSCRAMM family of cell-wall-anchored surface proteins of gram-positive cocci. Trends Microbiol..

[29] van Belkum A., Tassios P.T., Dijkshoorn L., Haeggman S., Cookson B., Fry N.K., Fussing V., Green J., Feil E., Gerner-Smidt P. (2007). Guidelines for the validation and application of typing methods for use in bacterial epidemiology. Clin. Microbiol. Infect..

[30] Ramadan A. (2022). A Bacterial typing methods from past to present: A comprehensive overview. Gene Rep..

[31] Harper J.J., Tilse M.H. (1991). Biotypes of *Haemophilus influenzae* that are associated with noninvasive infections. J. Clin. Microbiol..

[32] Gergova R.T., Tsitou V.M.S., Mitov I.G. (2019). Molecular-genetic method for fast direct detection of *Staphylococcus Aureus* and methicillin resistance in blood cultures and punctures. Folia Med..

[33] Gergova R., Muhtarova A., Petrova G., Gergov S., Dikova M. (2018). Comparison of cultural, immunological and new PCR techniques for detection of *Streptococcus pyogenes*. Comptes Rendus Acad. Bulg. Sci..

[34] Jin P., Xiao M., Kong F., Oftadeh S., Zhou F., Liu C., Gilbert G.L. (2009). Simple, accurate, serotype-specific PCR assay to differentiate *Streptococcus pneumoniae* serotypes 6A, 6B, and 6C. J. Clin. Microbiol..

[35] Davis G.S., Sandstedt S.A., Patel M., Marrs C.F., Gilsdorf J.R. (2011). Use of *bexB* to detect the capsule locus in *Haemophilus influenzae*. J. Clin. Microbiol..

[36] Falla T.J., Crook D., Brophy L., Maskell D., Kroll J.S., Moxon E.R. (1994). PCR for capsular typing of *Haemophilus influenzae*. J. Clin. Microbiol..

[37] Gergova R.T., Iankov I.D., Haralambieva I.H., Mitov I.G. (2007). Bactericidal monoclonal antibody against *Moraxella catarrhalis* lipooligosaccharide cross-reacts with *Haemophilus* spp.. Curr. Microbiol..

[38] Aguiar S., Serrano I., Pinto F., Melo-Christino J., Ramirez M. (2008). The presence of the pilus locus is a clonal property among pneumococcal invasive isolates. BMC Microbiol..

[39] Bagnoli F., Moschioni M., Donati C., Dimitrovska V., Ferlenghi I., Facciotti C., Muzzi A., Giusti F., Emolo C., Sinisi A. (2008). A second pilus type in *Streptococcus pneumoniae* is prevalent in emerging serotypes and mediates adhesion to host cells. J. Bacteriol..

[40] Montanaro L., Arciola C.R., Borsetti E., Brigotti M., Baldassarri L. (1998). A polymerase chain reaction (PCR) method for the identification of collagen adhesin gene (CNA) in Staphylococcus-induced prosthesis infections. New Microbiol..

[41] Pereira D.R.R., Pereira M.R., Pereira M.B.R., Costa S.S., Mott M.P., Cantarelli V. (2023). Otopathogens in the middle ear and nasopharynx of children with recurrent acute otitis media. Int. J. Pediatr. Otorhinolaryngol..

[42] Paton J.C., Trappetti C. (2019). *Streptococcus pneumoniae* capsular polysaccharide. Microbiol. Spectr..

[43] Ganaie F.A., Saad J.S., Lo S.W., McGee L., van Tonder A.J., Hawkins P.A., Calix J.J., Bentley S.D., Nahm M.H. (2023). Novel pneumococcal capsule type 33E results from the inactivation of glycosyltransferase WciE in vaccine type 33F. J. Biol. Chem..

[44] Alexandrova A.S., Boyanov V.S., Mihova K.Y., Hristova P.M., Hitkova H.Y., Marteva-Proevska Y., Gergova R.T. (2025). Population genetic structure of invasive and non-invasive *Streptococcus pneumoniae* isolates after fifteen years of routine PCV10 vaccination in Bulgaria. Int. J. Mol. Sci..

[45] Ekinci E., Van Heirstraeten L., Willen L., Desmet S., Wouters I., Vermeulen H., Lammens C., Goossens H., Van Damme P., Verhaegen J. (2023). Serotype 19A and 6C account for one-third of pneumococcal carriage among Belgian day-care children four years after a shift to a lower-valent PCV. J. Pediatr. Infect. Dis. Soc..

[46] Chen C.H., Chen C.L., Su L.H., Chen C.J., Tsai M.H., Chiu C.H. (2025). The microbiological characteristics and diagnosis of *Streptococcus pneumoniae* infection in the conjugate vaccine era. Hum. Vaccin. Immunother..

[47] Izurieta P., AbdelGhany M., Borys D. (2025). Serotype distribution of invasive and non-invasive pneumococcal disease in children ≤5 years of age following the introduction of 10- and 13-valent pneumococcal conjugate vaccines in infant national immunization programs: A systematic literature review. Front. Public Health.

[48] Bano S., Hassan N., Rafiq M., Hassan F., Rehman M., Iqbal N., Ali H., Hasan F., Kang Y.-Q. (2023). Biofilms as battlefield armor for bacteria against antibiotics: Challenges and combating strategies. Microorganisms.

[49] Zafer M.M., Mohamed G.A., Ibrahim S.R.M., Ghosh S., Bornman C., Elfaky M.A. (2024). Biofilm-mediated infections by multidrug-resistant microbes: A comprehensive exploration and forward perspectives. Arch. Microbiol..

[50] Quesada M.G., Peterson M.E., Bennett J.C., Hayford K., Zeger S.L., Yang Y., Hetrich M.K., Feikin D.R., Cohen A.L., von Gottberg A. (2025). Serotype distribution of remaining invasive pneumococcal disease after extensive use of ten-valent and 13-valent pneumococcal conjugate vaccines (the PSERENADE project): A global surveillance analysis. Lancet Infect. Dis..

[51] Weiser J.N., Ferreira D.M., Paton J.C. (2018). *Streptococcus pneumoniae*: Transmission, colonization and invasion. Nat. Rev. Microbiol..

[52] Slack M.P.E., Cripps A.W., Grimwood K., Mackenzie G.A., Ulanova M. (2021). Invasive *Haemophilus influenzae* infections after 3 decades of Hib protein conjugate vaccine use. Clin. Microbiol. Rev..

[53] Huska B., Ulanova M. (2025). Inflammatory responses to non-typeable *Haemophilus influenzae* clinical isolates from invasive and non-invasive infections. Pathogens.

[54] Sadeghi-Aval P., Tsang R.S., Jamieson F.B., Ulanova M. (2013). Emergence of non-serotype b encapsulated *Haemophilus influenzae* as a cause of pediatric meningitis in northwestern Ontario. Can. J. Infect. Dis. Med. Microbiol..

[55] Reilly A.S., McElligott M., Mac Dermott Casement C., Drew R.J. (2022). *Haemophilus influenzae* type f in the post-*Haemophilus influenzae* type b vaccination era: A systematic review. J. Med. Microbiol..

[56] Revai K., Mamidi D., Chonmaitree T. (2008). Association of nasopharyngeal bacterial colonization during upper respiratory tract infection and the development of acute otitis media. Clin. Infect. Dis..

[57] Danne C., Dramsi S. (2012). Pili of gram-positive bacteria: Roles in host colonization. Res. Microbiol..

[58] Barocchi M.A., Ries J., Zogaj X., Hemsley C., Albiger B., Kanth A., Dahlberg S., Fernebro J., Moschioni M., Masignani V. (2006). A pneumococcal pilus influences virulence and host inflammatory responses. Proc. Natl. Acad. Sci. USA.

[59] Nelson A.L., Ries J., Bagnoli F., Dahlberg S., Fälker S., Rounioja S., Tschöp J., Morfeldt E., Ferlenghi I., Hilleringmann M. (2007). RrgA is a pilus-associated adhesin in *Streptococcus pneumoniae*. Mol. Microbiol..

[60] Xiao J., Su L., Huang S., Liu L., Ali K., Chen Z. (2023). Epidemic trends and biofilm formation mechanisms of *Haemophilus influenzae*: Insights into clinical implications and prevention strategies. Infect. Drug. Resist..

[61] Alexandrova A.S., Boyanov V.S., Mihova K.Y., Gergova R.T. (2024). Phylogenetic Lineages and Diseases Associated with *Moraxella catarrhalis* Isolates Recovered from Bulgarian Patients. Int. J. Mol. Sci..

[62] Bernstein J.M., Reddy M. (2000). Bacteria-mucin interaction in the upper aerodigestive tract shows striking heterogeneity: Implications in otitis media, rhinosinusitis, and pneumonia. Otolaryngol. Head Neck Surg..

[63] Murphy T.F., Brauer A.L., Yuskiw N., Hiltke T.J. (2000). Antigenic structure of outer membrane protein E of *Moraxella catarrhalis* and construction and characterization of mutants. Infect. Immun..

[64] Akgul G., Erturk A., Turkoz M., Turan T., Ichinose A., Nagatake T., Ahmed K. (2005). Role of lipooligosaccharide in the attachment of *Moraxella catarrhalis* to human pharyngeal epithelial cells. Microbiol. Immunol..

[65] Zhao N., Ren H., Deng J., Du Y., Li Q., Zhou P., Zhou H., Jiang X., Qin T. (2022). Genotypic and Phenotypic Characteristics of *Moraxella catarrhalis* from Patients and Healthy Asymptomatic Participants among Preschool Children. Pathogens.

[66] Holm M.M., Vanlerberg S.L., Foley I.M., Sledjeski D.D., Lafontaine E.R. (2004). The *Moraxella catarrhalis* porin-like outer membrane protein CD is an adhesin for human lung cells. Infect. Immun..

[67] Gonzalez-Reyes M., Ramos-Tapia I., Ugalde J.A. (2025). A global perspective on the genomics of *Moraxella catarrhalis*. Microb. Genom..

[68] de Vries S.P.W., Bootsma H.J., Hays J.P., Hermans P.W.M. (2009). Molecular aspects of *Moraxella catarrhalis* pathogenesis. Microbiol. Mol. Biol. Rev..

[69] Castro S.A., Dorfmueller H.C. (2021). A brief review on Group A Streptococcus pathogenesis and vaccine development. R. Soc. Open Sci..

[70] Thacharodi A., Hassan S., Vithlani A., Ahmed T., Kavish S., Geli Blacknell N.M., Alqahtani A., Pugazhendhi A. (2024). The burden of group A *Streptococcus* (GAS) infections: The challenge continues in the twenty-first century. iScience.

[71] Hasegawa T., Minami M., Okamoto A., Tatsuno I., Isaka M., Ohta M. (2010). Characterization of a virulence-associated and cell-wall-located DNase of *Streptococcus pyogenes*. Microbiology.

[72] Remmington A., Turner C.E. (2018). The DNases of pathogenic Lancefield streptococci. Microbiology.

[73] Aziz R.K., Ismail S.A., Park H.W., Kotb M. (2004). Post-proteomic identification of a novel phage-encoded streptodornase, Sda1, in invasive M1T1 Streptococcus pyogenes. Mol. Microbiol..

